# Carbapenem-producing *Enterobacteriaceae* in mothers and newborns in southeast Gabon, 2022

**DOI:** 10.3389/fcimb.2024.1341161

**Published:** 2024-02-08

**Authors:** Sandra Dos Santos, Mesmin Moussounda, Moussa Togola, Evelyne Avoune Nguema, Christiane Matteya, Michelle Bignoumba, Richard Onanga, Jean-Bernard Lekana-Douki, Patrice François, Nathalie van der Mee-Marquet

**Affiliations:** ^1^ Centre d’appui pour la prévention des infections associées aux soins en région Centre Val de Loire, Hôpital Bretonneau, Centre Hospitalier Régional Universitaire, Tours, France; ^2^ Laboratoire d'Analyses médicales, Hôpital d'Instruction des Armées Omar Bongo Ondimba, Libreville, Gabon; ^3^ Equipe « bactéries et risque materno-fœtal », Unité Mixte de Recherche (UMR) 1282, Infectiologie et Santé Publique INRAE, Tours, France; ^4^ Maternité, Centre Hospitalier Régional Amissa Bongo, Franceville, Gabon; ^5^ Service de Néonatalogie, Centre Hospitalier Régional Amissa Bongo, Franceville, Gabon; ^6^ Centre Interdisciplinaire de Recherches Médicales de Franceville, Franceville, Gabon; ^7^ Laboratoire de Recherche Génomique, Hôpitaux Universitaires et faculté de Médecine de Genève, Geneva, Switzerland

**Keywords:** carbapenem-resistant *Enterobacteriaceae*, carbapenemase, third generation cephalosporins resistance, newborn, Gabon

## Abstract

**Introduction:**

Infections caused by carbapenemase-producing Enterobacteriaceae (CPE) pose a significant threat, leading to severe morbidity and mortality among newborns.

**Methods:**

This study, conducted at Franceville hospital's maternity and neonatology wards from February 22nd to June 20th, 2022, investigated the prevalence of CPE in 197 parturients and 203 newborns. Rectal swabs were taken from parturients before delivery and from newborns 30 minutes after birth. Blood culture samples were collected if signs of infection were observed in newborns during a 28-day follow-up. A total of 152 environmental samples were obtained, comprising 18 from sinks, 14 from incubators, 27 from cradles, 39 from maternal beds, 14 from tables and desks, four from the two baby scales and 36 from bedside furniture.

**Results:**

None of the 203 newborns were found to be CPE carriers 30 minutes after delivery. CPE carriage was found in 4.6% of mothers. When comparing colonized and uncolonized parturients, well-established risk factors for CPE carriage, such as recent hospitalization and antibiotic therapy, were more frequently observed among CPE carriers (33.3 vs 10.6% for hospitalization in the past 15 days; 55.5 vs 30.3% for hospitalization during pregnancy, and 55.5 vs 35.1% for antibiotic therapy during pregnancy). Notably, the prevalence of treatment with amoxicillin and clavulanic acid was 44.4% in CPE carriers compared to 17.0% in non-carriers. The incidence density of CPE-associated bloodstream infection was 0.49 per 100 newborns, accounting for a fatal case of CPE-associated bacteremia identified in one of the 203 newborns. Seven environmental samples returned positive for CPE (5 sinks and two pieces of furniture). Whole genome sequencing, performed on the 25 CPE isolates, revealed isolates carrying blaNDM-7 (n=10), blaNDM-5 (n=3), blaOXA181 (n=10), blaOXA48 (n=2) or blaOXA244 (n=1), along with genetic traits associated with the ability to cause severe and difficult-to-treat infections in newborns. Core genome comparison revealed nine CPE belonging to three international high-risk clones: E. coli ST410 (four mothers and a sink), two E. coli ST167 (a mother and a piece of furniture), and K. pneumoniae ST307 (a sink and a piece of furniture), with highly similar genetic backgrounds shared by maternal and environmental isolates, suggesting maternal contamination originating from the environment.

**Discussion:**

Our study reveals key findings may guide the implementation of infection control measures to prevent nosocomial infections in newborns: the prevalence of CPE carriage in one out of 20 parturients, an infection occurring in one out of 400 newborns, substantial contamination of the care environment, clinical and environmental CPE isolates possessing genetic traits associated with the ability to cause severe and challenging infections, and clonal relationships between clinical and environmental isolates suggesting CPE spread within the wards, likely contributing to the acquisition and colonization of CPE by parturients during pregnancy.

## 1 Introduction

Infections caused by carbapenemase-producing *Enterobacteriaceae* (CPE) pose a significant threat, leading to severe morbidity and mortality among patients hospitalized in intensive care units and newborns (Dautzenberg et al., 2015).

Two major factors contribute to the colonization and infection of newborns by such agents (Bulabula et al., 2017; Bulabula et al., 2020): firstly, the colonization of mothers by CPE, facilitated by the inappropriate use of broad-spectrum antibiotics and the contamination of the parturient’s environment by CPE; and secondly, the contamination of the neonatal environment. The dissemination of the CPE in maternity and neonatal wards may be facilitated under specific circumstances, including instances where systematic hand hygiene is not observed immediately before caring for parturients and newborns, when the disinfection of reusable medical equipment is inadequate, if the implementation of environmental cleaning protocols falls short, and/or when healthcare professionals lack sufficient training in good hygiene practices.

Despite an increasing number of reports on CPE in Central Africa (Moussounda et al., 2017; Singh et al., 2018; Okomo et al., 2019; Mouanga-Ndzime et al., 2023), there is a notable gap in the literature concerning epidemiological studies focused on the carriage and infection rates of CPE among mothers and newborns in maternity hospitals, as well as the contamination of the care environment by these pathogens. In 2016, a prevalence study conducted at the military hospital in Libreville identified the presence of *K. pneumoniae bla*_NDM-7_ in three out of seven newborns present. However, no investigation was conducted to explore the mechanism of acquisition of this CPE by the newborns (Moussounda et al., 2017).

To address this knowledge gap and aid in the better selection of probabilistic treatments to manage locally acquired neonatal infections, we conducted an epidemiological study at the regional hospital in Franceville, the capital of the Haut-Ogooué province (with a population of 250,799 inhabitants). Our study aimed to determine the rates of CPE carriage among mothers and newborns at the time of delivery and to assess the incidence of neonatal bloodstream infections caused by these pathogens. Additionally, we explored the risk factors associated with maternal carriage of CPE and the contamination of the hospital environment. As part of our investigation, we utilized whole-genome sequencing to gain insights into the genetic diversity, virulome, resistome, and plasmid content of clinical and environmental CPE isolates collected during the study.

## 2 Materials and methods

### 2.1 Study design

The study was conducted between February 22nd and June 20th, 2022, at the maternity ward and neonatology ward of Franceville Hospital (**Supplementary Figure 1**). The study population included all consecutive parturients who gave birth during this period, along with their newborns.

### 2.2 Data collection

For mothers, we collected gathered information on age and clinical data, including parity, underlying pathologies, gynecological-obstetrical history, and any previous hospitalization or antibiotic therapy within the last 6 months. For newborns, the collected data included sex, gestational age, birth weight, use of invasive devices, and antibiotic usage since reaching 28 days of age.

### 2.3 Microbiological study

#### 2.3.1 Clinical Samples

Rectal swabs were collected using swabs with Amies-type preservation medium (Copan Italia SPA, Italy) immediately before delivery for mothers and 30 minutes after birth for newborns. In the presence of clinical signs suggestive of infection were present during the 28-day follow-up of the newborns, blood culture samples were taken (BacTAlert PF Plus pediatric bottle; bioMérieux, Marcy-l’Étoile, France) before initiating antibiotic therapy.

#### 2.3.2 Environmental samples

The maternity ward comprises a delivery room with four birthing rooms, a pre-delivery room with three separate spaces, two cleansing rooms for cleaning and desinfecting reusable equipment, eight bedrooms, two duty rooms, a medical office, and a locker room. The neonatology ward includes a main room accommodating six incubators and six cribs, a bedroom, and a desk room for medical assessments (**Supplementary Figure 1**). The samples were taken from all water points used in the facilities, as well as a significant number of surfaces in close proximity to parturients and newborns, especially those believed to be regularly touched by healthcare professionals during the care provided to parturients or newborns. We sampled the sinks (labeled S1 to S10) at all tap water points from the wards, along with 106 surfaces in the immediate proximity of newborn care. This included all incubators (n=7; I1-I7), all cradles (n=26; C1-C26), all maternal beds (n=33; B1-B33), all tables and desks (n=9; D1-D9), the two baby scales and 29 bedside furniture pieces.

#### 2.3.3 CPE detection and characterization

Blood cultures were all plated on CHROMagar Orientation medium (CHROMagar, Paris, France) on day 7. Rectal and environmental swabs were screened for third-generation cephalosporin- and carbapenem-resistant *Enterobacteriaceae* using CHROMagar ESBL and mSuperCARBA chromogenic agar plates (CHROMagar, Paris, France). After incubation at 37°C for 24 hours under aerobic conditions, we identified each colony morphotype using Matrix-assisted laser desorption-ionization-time–of-flight mass spectrometry (microflex LT MALDI-TOF, MBT Compass software version 4.2.100.19; Bruker Daltonics, France). All *Enterobacteriaceae* underwent antibiotic sensitivity testing (EUCAST 2022), including Ertapenem Minimum Inhibitory Concentration (MIC) (E-test strip; bioMérieux, Marcy-l’Étoile, France) and colistin MIC (colistin UMIC plates; Bruker Daltonics, France). Carbapenemase production was investigated when Ertapenem MIC > 0.5 mg/l using the immunochromatographic test RESIST-5 O.K.N.V.I (Coris BioConcept, Belgium) and confirmed using a PCR test for molecular detection of the genes encoding for the five carbapenemases of primary public health concern (*bla*_OXA48_, *bla*_NDM_, *bla*_KPC_, *bla*_VIM_ and *bla*_IMP_ genes) (Doyle et al., 2012). All CPE underwent whole-genome sequencing using next-generation sequencers (NGS). Purified genomic DNA (obtained using the DNeasy kit, Qiagen) for each strain was sequenced on the Illumina HiSeq (Illumina, San Diego, California), generating 100-base pairs paired-end reads and using barcodes following the Nextera XT kit (Illumina) per the manufacturer’s recommendations. Read quality was assessed using Trimmomatic v0.36 with the following parameters for paired end reads: SLIDINGWINDOW:10:30 and a minimum read length of 80. Genome assembly was performed using the SPADES v3.12.0 assembler. Assembled genomes and plasmids (identified using mlplasmid available at https://sarredondo.shinyapps.io/mlplasmids/) were annotated using the RAST server (https://rast.nmpdr.org/rast.cgi). Multilocus sequence typing analysis (*in silico*) was conducted using annotated genomes and submitted to the Center for Genomic Epidemiology database (available at: https://cge.food.dtu.dk/services/MLST/). The phylogenetic relationships of all isolates were investigated through genomic single-nucleotide polymorphism (SNP)–based analysis (CSIPhylogeny). Additional tools, available at the Center for Genomic Epidemiology (https://www.genomicepidemiology.org/ , were used for detecting antibiotic resistance (https://cge.food.dtu.dk/services/ResFinder/), mobile genetic elements (https://cge.food.dtu.dk/services/MobileElementFinder/), and virulence factors (https://cge.food.dtu.dk/services/VirulenceFinder/). All genome and plasmid sequences have been deposited in the European Nucleotide Archive database under accession number PRJEB66188 ERP151258.

### 2.4 Statistical analysis

We used Pearson’s chi-square test or Fisher’s exact test to compare categorical variables.

### 2.5 Ethical considerations

The study protocol was developed in collaboration with the heads of the two wards (medical, midwives, nurses). Following approval from the Ministry of Health, research authorization was granted by the Director General of the hospital. Each parturient received an individual letter of information and provided consent before enrollment. Anonymity and confidentiality were maintained for all collected data.

## 3 Results

The study involved 197 consecutive mothers and their 203 newborns, recruited over a two-month period. Key characteristics of the population are presented in **Table 1**. Recent hospitalization and antibiotic therapy were reported in 31.5% and 36.0% of the parturients, respectively. Deliveries were predominantly vaginal (72.1%). Five *in-utero* deaths were recorded. Among newborns, 5.9% had a birth weight less than 2,000 grams, 73.9% displayed signs of infection and received ceftriaxone (for 5-7 days, 20-50 mg/kg/24h) and gentamicin (for 3 days, 3-5 mg/kg/24h). After a 28-day follow-up, accounting for five stillbirths, the mortality rate within the studied population was 4.9%.

Table 1Characteristics of 197 parturients and 203 newborns based on maternal CPE-carriage status at delivery.

**Table:** 

	All	CPE-carriage status		p
		carriers	non carriers	
Mothers	197	9	188	
Median age (year)	25	23	25	
Parity (one or more previous children) (%)	133 (67.5)	7 (77.8)	126 (67.0)	0.757
Cesarian birth way (%)	55 (27.9)	3 (33.3)	52 (27.7)	1
Hospitalization in the past 15 days (%)	23 (11.7)	3 (33.3)	20 (10.6)	0.123
Hospitalization during pregnancy (%)	62 (31.5)	5 (55.5)	57 (30.3)	0.220
Antibiotherapy during pregnancy (%)	71 (36.0)	5 (55.5)	66 (35.1)	0.372
Amoxicillin + clavulanic acid (%)	36 (18.3)	4 (44.4)	32 (17.0)	0.101

**Table:** 

Newborns	All	From mother with CPE-carriage status		
		carriers	non carriers	
	203	9	194	
Median birth weight (g)	2900	2550	2900	
Birth weight < 2,000 (%)	12 (5.9)	1 (11.1)	11 (5.7)	1
Median gestational age (WA*)	39	37	39	
Gestational age < 30 WA (%)	4 (2.0)	1 (11.1)	3 (1.5)	1
Signs of infection at delivery	150 (73.9)	6 (66.7)	144 (74.2)	0.907
Mucocutaneous disorders	120 (59.1)	4 (44.4)	116 (59.8)	0.569
Respiratory disorders	37 (18.2)	2 (22.2)	35 (18.0)	1
Elevated C-reactiv protein	16 (10.7)	2 (22.2)	14 (7.2)	0.317
Fever	4 (2.0)	0	4 (2.1)	1
CPE-associated infection	1 (0.5)	0	1 (0.5)	1
*In utero* death (%)	5 (2.5)	0	5 (2.6)	1
Death within 28 days of life (%)	5 (2.5)	1 (11.1)	4 (2.1)	0.556

*WA weeks of amenorrhea.

### 3.1 CPE detection

#### 3.1.1 Maternal CPE carriage at delivery

Among the 197 parturients, nine were identified as CPE carriers (4.6%; **Table 1**). The nine mothers carrying CPE all carried *E. coli*. Three mothers carried two CPE, resulting in a total of 12 CPE strains (**Table 2**). *E. coli* was the predominant species, constituting nine strains (75.0%) including six carrying *bla*_OXA-181_ (four ST140, one ST38, and one ST5232 co-carrying *bla*_OXA-244_), one ST167 carrying *bla*_NDM-5_, and two ST11155 (one carrying *bla*_NDM-7_ and one carrying *bla*_OXA-48_). The remaining maternal CPE consisted of two ST204 *C. freundii* carrying *bla*_NDM-7_ (16.7%) and one ST1037 *K. pneumoniae* carrying *bla*_OXA-181_ (8.3%). Additionally, a mother carried carbapenem-resistant ST45 *K. pneumoniae*, with no carbapenemase production. In comparison to non-carriers, CPE carriers had a higher history of recent hospitalization (55.5 vs 30.3%) and recent antibiotic therapy (55.5 vs 35.1%); however, these differences remained non-significant (**Table 1**).

**Table 2:** Characteristics of the 25 clinical and environmental carbapenemase-producing *Enterobacteriaceae*.

	CPE strains from											
	colonized mothers				Infected newborn				Environmental samples			
	Mothers	Strain code	MLST	Carb. genes*	Newborn	Strain code	MLST	Carb. genes*	Samples	Strain code	MLST	Carb. genes*
*E. coli*	M117	756_S7	11155	*bla*_OXA-48_								
	M24	623_S2	38	*bla*_OXA-181_								
	M37	753_S11	410	*bla*_OXA-181_					Sink S1 (delivery room)	792_S10	410	*bla*_OXA-181_
	M25B	751a_S4	410	*bla*_OXA-181_								
	M35	748a_S3	410	*bla*_OXA-181_								
	M140	611_S1	410	*bla*_OXA-181_								
	M19	754_S5	5232	*bla*_OXA-181_ *bla*_OXA-244_								
	M42	755_S6	167	*bla*_NDM-5_	N41	774_S9	2083	*bla*_NDM-5_	A meubel in bedroom B9	711ec	167	*bla*_NDM-5_
	M118	757a_S8	11155	*bla*_NDM-7_								
*C. freundii*	M37	639_S18	204	*bla*_NDM-7_					Incubator I7 (delivery room)	715_S19	579	*bla*_NDM-7_
	M118	757b_S20	204	*bla*_NDM-7_					A meubel in bedroom B9	711cf	91	*bla*_NDM-7_
									Sink S2 (cleansing room, delivery unit)	801_S24	91	*bla*_NDM-7_
									Sink S6 (cleansing room, maternity ward)	800_S23	91	*bla*_NDM-7_
*K. pneumoniae*	M35	748b_S15	1037	*bla*_OXA-181_					A meubel in bedroom B9	711_S13	307	*bla*_OXA-181_
									Sink S6 (cleansing room, maternity ward)	713_S14	307	*bla*_OXA-181_
									Sink S5 (bedroom B6)	700_S12	22	*bla*_OXA-48_
*E. cloacae*									Sink S1 (delivery room)	794_S22	45	*bla*_NDM-7_
									Sink S2 (cleansing room, delivery unit)	789_S21	1065	*bla*_NDM-7_
*Leclercia sp.*									Sink S3 (cleansing room, delivery unit)	706_S25	ND	*bla*_NDM-7_

*Carb.genes, carbapenemase genes.

#### 3.1.2 Neonatal CPE-associated bacteremia

None of the 203 newborns were found to be CPE carriers 30 minutes after delivery. The follow-up of the newborns up to 28 days of life revealed bacteremia in 16 neonates (7.9%), predominantly during the first week of life (81.2%). None of the infected newborns had a mother colonized by a CPE at the time of delivery. Among the 16 newborns, ten were infected with *Enterobacteriaceae*, including one CPE identified as ST2083 *E. coli* carrying *bla*_NDM-5_ (**Table 2**). Consequently, the incidence density of CPE-associated bloodstream infection was 0.49 per 100 newborns. The infected newborn was a premature girl with a gestational age of 32 weeks of amenorrhea and a birth weight of 2,150g. Her 24-year-old mother had no history of recent hospitalization or antibiotic therapy, and was not identified as CPE carrier. The delivery was conducted by caesarian section. Considering respiratory distress beginning after the first hours of life and an elevated C-reactiv protein, antibiotic therapy was initiated with a combination of ceftriaxone and gentamicin just before obtaining blood cultures. Unfortunately, the newborn passed away after five days.

#### 3.1.3 Environmental contamination

A total of 152 samples were collected: 18 from sinks, 14 from incubators, 27 from cradles, 39 from maternal beds, 14 from tables and desks, four from the two baby scales and 36 from bedside furniture. Out of the 152 samples tested, seven returned positive for CPE (**Table 2**): the sink in bedroom B6 (S5), a piece of furniture in bedroom B9, the sink (S1) and the incubator (I7) in the delivery room, the two sinks in the cleansing room of the delivery unit (S2 and S3) and the sink S6 in the cleansing room of the maternity ward. Twelve CPE were identified, carrying *bla*_NDM-7_ present in seven cases (58.3%), including two *E. coli* (16.7%; one ST167 carrying *bla*_NDM-5_and one ST140 carrying *bla*_OXA-181_), four *C. freundii* carrying *bla*_NDM-7_ (33.3%; three of ST91 and one ST579), three *K. pneumoniae* (25.0%; one ST22 carrying *bla*_OXA-48_ and two ST307 carrying *bla*_OXA-181_), two *E. cloacae* carrying *bla*_NDM-7_ (16.7%; one ST45 and one ST1065), and one *Leclercia* sp carrying *bla*_NDM-7_ (8.3%).

**Figure 1:**
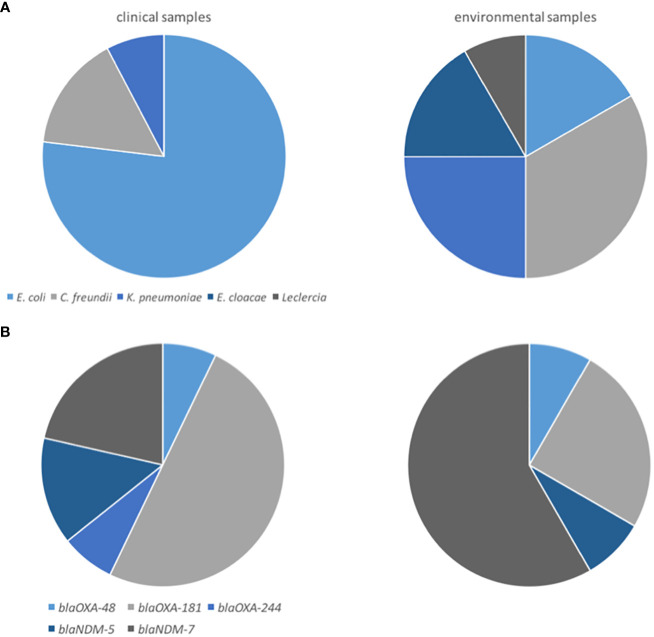
Distribution of the CPE by species **(A)**, and by carbapenemase gene **(B)**, stratified across strain origin.

Overall, we disposed encountered 13 clinical and 12 environmental CPE, representing five species: *E. coli* (12; 48.0%), six *C. freundii* (24.0%), *K. pneumoniae* (4; 16.0%), two *E. cloacae* (8.0%), and one *Leclercia* sp (4.0%) (**Table 2**). *E. coli* isolates were more prevalent among clinical isolates (76.9% of *E. coli* among clinical isolates vs. 16.7% among environmental isolates; p=0.009) (**Figure 1A**). The carbapenemase-associated genes detected in the 25 CPE isolates included *bla*_NDM-7_ in ten isolates (three maternal and seven environmental), *bla*_OXA-181_ in ten isolates (seven maternal and three environmental), *bla*_NDM-5_ in three isolates (one maternal, one neonatal and one environmental), *bla*_OXA-48_ in two isolates (one maternal and one environmental), and *bla*_OXA-244_ (one maternal isolate) (**Table 2**, **Figure 1B**). The genetic diversity of the CPE was limited, with five ST410 *E. coli* (four maternal colonizing isolates and an isolate from the sink S1), three environmental ST91 *C. freundii* isolated from sinks S2 and S6 and furniture in bedroom B9, two maternal colonizing ST11155 *E. coli*, two ST167 *E. coli* (a maternal colonizing isolate and an isolate from furniture in bedroom B9), two maternal colonizing ST204 *C. freundii*, and two environmental ST307 *K. pneumoniae* isolated from the sink S6 and furniture in bedroom B9.

### 3.2 Whole genome sequencing

We characterized the 27 available carbapenem-resistant isolates, including the 25 CPE and the two carbapenem-resistant ST45 *K. pneumoniae* with no carbapenemase production.

#### 3.2.1 Core genome comparison of the CPE

**Figure 2:**
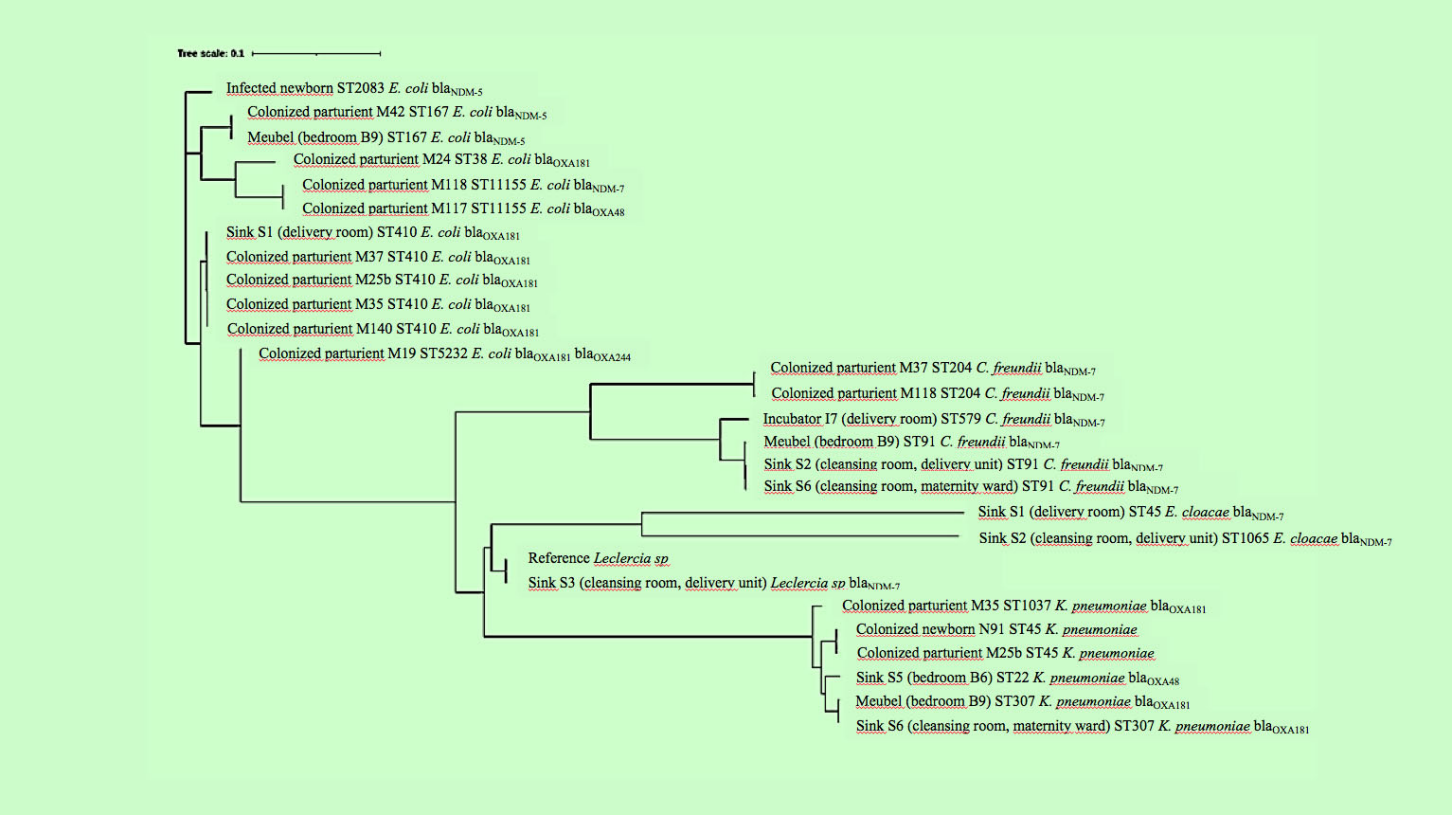
Genetic diversity of the 27 *Enterobacteriaceae* studied.

Revealed clusters of highly similar strains (**Figure 2**; **Supplementary Table 1**): the ST410 *E. coli* recovered from four mothers and a sink, the two ST167 *E. coli* recovered from a mother and a piece of furniture, the two maternal ST11155 *E. coli*, the two maternal ST204 *C. freundii*, the three environmental ST91 *C. freundii*, the two environmental ST307 *K. pneumoniae*, and the two ST45 *K. pneumoniae* colonizing a mother and her newborn.

#### 3.2.2 The resistome study

Revealed that most isolates carried genes associated with resistance to fluoroquinolones (88.9%), aminoglycosides (77.8%), sulfonamides (77.8%), trimethoprim (77.8%), tetracyclines (74.1%), and disinfectants (77.8%) (**Supplementary Table 2**). Elevated MICs for colistin were observed in two *C. freundii* isolates (MIC colistin=4 mg/L) and one *E. cloacae* isolate (MIC colistin=64 mg/L), with no detected carriage of *mcr* genes. The *K. pneumoniae* isolates colonizing M25B and N91, lacking carbapenemase-associated genes, contained a chromosome-encoded gene with a 66% homology to one encoding a metallo-beta-lactamase superfamily protein. This gene confers resistance to carbapenems and may explain the observed diminished susceptibility to carbapenems (Bebrone, 2007).

#### 3.2.3 The virulome study

Revealed that the majority of *E. coli strains* carried genes encoding adhesins, hemolysins, siderophores and the ferric uptake regulator gene *irp2* (Tu et al., 2016) (**Supplementary Table 3**). Additionally, they exhibited the coiled surface structure curlin, involved in inert surface colonization and biofilm formation (*csgA*), the glutamate decarboxylase *gad* gene (Grant et al., 2001), which is a marker of pathogenic *E. coli*, a lipoprotein (nlpL), the *iss* gene, contributing to serum survival in virulent septicemic *E. coli* strains (Biran et al., 2021), and the tellurite resistance gene *terC*, which has been associated with colicine resistance and pathogenicity (Turkovicova et al., 2016). Among the six *K. pneumoniae* isolates, genes encoding adhesins fimHb and mrkA, were observed, as well as the *iutA* gene encoding a siderophore, the *clpK1* gene encoding a protein involved in resistance to thermal stress, and the *traT* gene associated with serum resistance.

#### 3.2.4 The plasmid study

**Figure 3:**
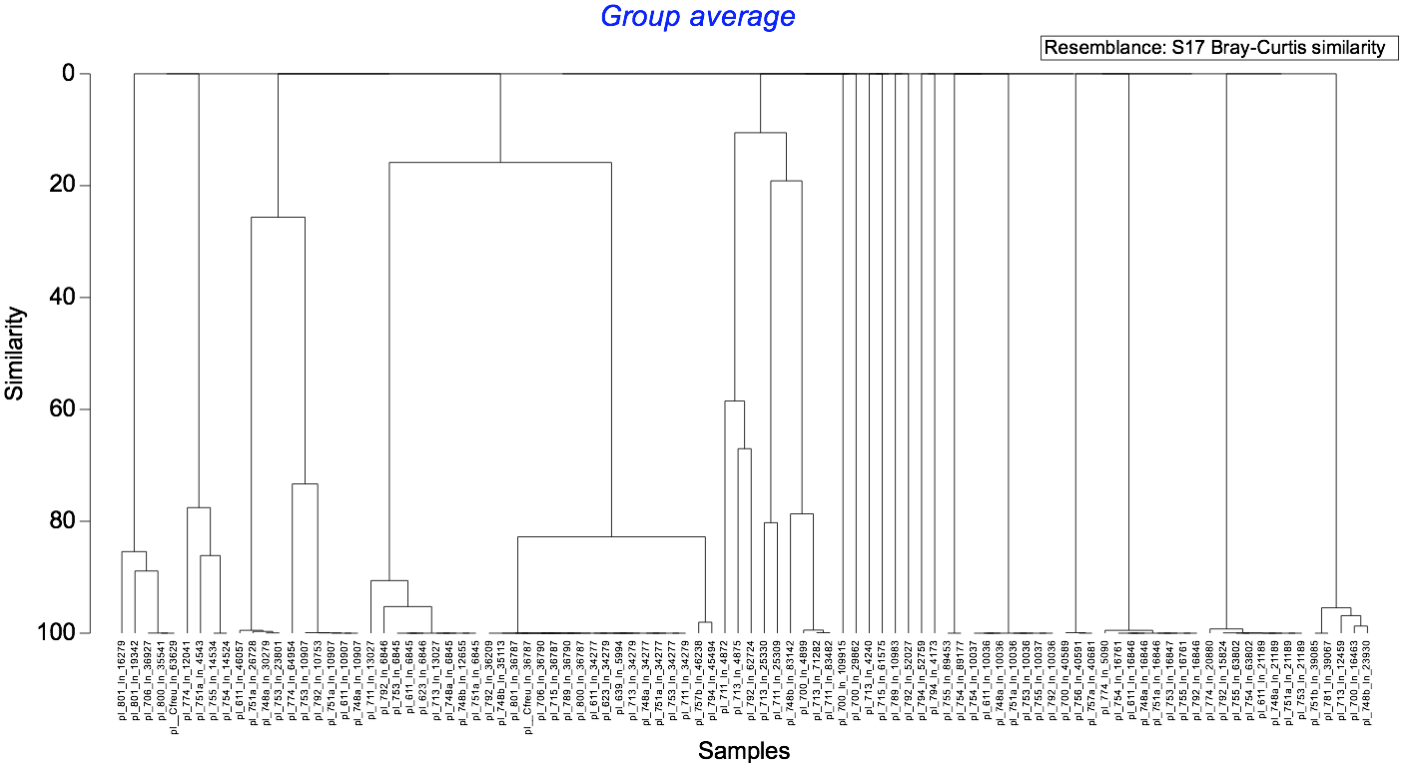
Genetic diversity of the plasmids carried by the 27 *Enterobacteriaceae* studied.

Identified 96 plasmids within the 27 genomes, with 88 of them clustered into 15 groups of highly similar plasmids (**Figure 3**). The number of plasmid sequences per bacterial genome ranged from zero to eight, with a median value of three. Plasmid sequences were more prevalent among *E. coli* isolates (median value of five plasmids per genome) and *K. pneumoniae* (median value of 4.5) compared to the remaining three species (median value of two plasmids per genome) (p=0.018). Among the 27 studied isolates, IncX3 plasmids were present in 18 CPE, and ColKP3 plasmids were carried by all the CPE carrying *bla*_OXA-181_. The isolates with highly similar core genomes exhibited similar plasmid content.

## 4 Discussion

Our study, encompassing a significant population of Gabonese parturients and newborns, brought a detailed snapshot of the current situation in the maternity setting at the regional hospital of Franceville.

First and foremost, to the best of our knowledge, this study represents the initial investigation into the carriage rate of CPE in Gabonese mothers at the time of delivery. Similar to findings reported in Algeria (4.1% in mothers; Mairi et al., 2019), our study explores the occurrence of colonizing CPE in 4.6% of the parturients at the time of delivery (i.e., one out of 20 parturients). This result, contrasting with the 2016 study at the hospital in Libreville [where none of 26 parturients tested were found to be CPE carriers in a one-day prevalence study (Moussounda et al., 2017)], suggests an increasing prevalence of CPE carriage among the young population of Gabonese parturients.

Of the nine colonized parturients, CPE belonged to the *E. coli* species, with three being co-colonized with either *C. freundii* or *K. pneumoniae*. The predominance of carbapenemase-producing *E. coli* is a matter of concern, because unlike *K. pneumoniae*, which is primarily a nosocomial pathogen, *E. coli* isolates are responsible for both community- and hospital-acquired infections, thus raising the fear of CPE dissemination in the community (Gauthier et al., 2018).

When comparing colonized and uncolonized parturients, well-established risk factors for CPE carriage, such as recent hospitalization and antibiotic therapy, were more frequently observed with CPE carriers. Notably, the prevalence of treatment with amoxicillin and clavulanic acid was found in 44.4% of the CPE carriers and 17.0% of non-carriers. However, the differences between the two groups of parturients remained non-significant, likely due to the insufficient number of parturients included and the high prevalence of hospitalization (31.5%) and antibiotic therapy (36.0%) history during pregnancy for the entire population of parturients cared in the hospital. Additionally, an *E. coli* strain belonging to the ST410 clone was detected in half of the carriers of CPE among the mothers. This clone has been extensively described in epidemiological studies involving multiresistant *E. coli* strains in animals, suggesting its dissemination in the food chain (López-Cerero et al., 2011). It would have been interesting to investigate the presence of this clone in the food consumed by the residents of Franceville. However, this aspect was not explored in our study.

Second, none of the newborns with a CPE carrier mother was found colonized by CPE during the first hour of life, and none of the newborns with a CPE carrier mother was infected by the maternal CPE before 28 days of life. Furthermore, the mother of the sole newborn diagnosed with CPE-associated bacteremia was not herself a carrier of CPE upon arrival at the maternity ward for delivery. Maternal carriage of multiresistant *Enterobacteriaceae* is, however, a well-described risk factor for neonatal acquisition; the neonate may acquire the maternal flora, favored by the proximity between mother and newborn. In our study, the CPE test was conducted after 30 minutes of life, a timeframe likely too short to detect neonatal acquisition from the mother. Further tests would have been necessary to demonstrate neonatal acquisition of maternal CPE. Our study was not designed to address this specific point.

We report an incidence density of CPE-associated bloodstream infection (0.49 per 100 newborns) in the examined Gabonese newborns. While the infection linked to *E. coli* carrying *bla*_NDM-5_ likely contributed to the death of the premature newborn, our data do not definitively establish the exact role of CPE in the infant’s mortality; what is clear is that the antibiotic treatment administred to the baby, involving third-generation cephalosporin and gentamicin, was ineffective against the implicated CPE. As this CPE was not detected in the mother’s flora, but considering the evidence of contaminated sinks and surfaces in proximity to newborns, our findings suggest that the primary acquisition of CPE by the newborn may have originated from the environment during care. Unfortunately, and while two strains of CPE belonging to the *E. coli* species were isolated from environmental samples, the *E. coli* strain carrying *bla*_NDM-5_ responsible for the newborn’s bacteremia was not isolated from the environment. The investigation of likely sites of CPE acquisition would have required a much higher number of environmental samples, distributed regularly throughout the entire study duration. Unfortunately, our one-shot study was not designed for this purpose.

However, the WGS analysis revealed clinical and environmental CPE isolates with similar genetic backgrounds, such as the ST410 *E. coli* clone carrying *bla*_OXA-181_ (Roer et al., 2018) found in four parturients and a sink, the ST167 *E. coli* clone carrying *bla*_NDM-5_ (Garcia-Fernandez et al., 2020) found in a parturient and the environment. Investigations of outbreaks associated with CPE have demonstrated the ability of these bacteria to establish themselves in sinks, survive in biofilms, and contaminate hands and surfaces during care due to splashing effects, particularly when hygiene practices are inadequate (Roux et al., 2013; Decraene et al., 2018). These investigations primarily involve CPE belonging to *K. pneumoniae*, *C. freundii*, and *E. cloacae* complex, excluding *E. coli*. In our study, the observation of highly similar CPE isolates belonging to the *E. coli* species, both in maternal and environmental samples, suggests, though not conclusively, probable maternal contaminations originating from the hospital environment. Throughout the study, the examination of professional practices uncovered that practitioners lacked access to hand-rubbing solutions, and hand hygiene practices before providing care to newborns were not consistently observed (data not shown). In this context, it is plausible that parturients may have been exposed to CPE during pregnancy through manual handling, originating from their immediate environment. Additional investigations are currently underway in the two wards to delve into these aspects.

Out of the 25 CPE strains, most harbored genetic traits associated with antibiotic resistance and virulence. Nine belong to international high-risk clones, namely the ST410 (Roer et al., 2018) and ST167 (Garcia-Fernandez et al., 2020) *E. coli* clones, and the ST307 *K. pneumoniae* clone, which have been associated with hospital outbreaks and have a high ability to spread in the hospital setting, in relation with their frequent carriage of hyperconjugative IncX3 and ColKP3 plasmids (Carfora et al., 2022). Additionally, we identified *E. coli* of ST2083, a clone associated with a fatal infection in a newborn in Tanzania (Manyahi et al., 2022), and ST38, responsible for extra-intestinal infections in humans (Biez et al., 2022). The identification, in close proximity to occasionally fragile newborns, of numerous mothers carrying potentially virulent strains of CPE resistant to the antibiotics currently used for treating infected newborns in this facility, along with a notable contamination of the care environment, underscores a concerning situation that warrants immediate attention and the implementation of improvement measures.

## 5 Conclusion

Our study highlights five key findings: the prevalence of CPE carriage in one out of 20 parturients, the occurrence of an infection in one out of 400 newborns, substantial contamination of the care environment in the maternity and neonatology wards of the hospital in Franceville, clinical and environmental CPE isolates possessing genetic traits associated with the ability to cause severe and challenging infections in newborns, and clonal relationships between maternal and environmental isolates suggesting CPE spread within the two wards, likely contributing to the acquisition and colonization of CPE by parturients during pregnancy. To address this, we have initiated an intervention plan, which includes providing hand-rubbing alcoholic solutions, educating healthcare workers on hand hygiene before attending to mothers and newborns, removing contaminated sinks, and implementing daily disinfection of all water points. We plan to reassess the situation six months after the intervention to evaluate the impact of the infection control plan.

The replication of this study in other neonatology services could provide a precise understanding of the dissemination of CPE in these facilities. It may guide the implementation of infection control measures to prevent nosocomial infections in newborns and tailor empiric antibiotic treatments for infected newborns, if necessary.

## Data availability statement

The datasets presented in this study can be found in online repositories. The names of the repository/repositories and accession number(s) can be found below: https://www.ebi.ac.uk/ena, PRJEB66188 ERP151258.

## Ethics statement

The studies involving humans were approved by André NTCHORERET OLUSEGUN, Directeur Général du CHR Amissa BONGO sous couvert du Ministre de la Santé et des Affaires Sociales de la République Gabonaise. The studies were conducted in accordance with the local legislation and institutional requirements. Written informed consent for participation in this study was provided by the participants’ legal guardians/next of kin.

## Author contributions

SS: Conceptualization, Data curation, Formal analysis, Methodology, Project administration, Writing – original draft, Supervision, Validation. MM: Data curation, Formal analysis, Investigation, Methodology, Conceptualization, Writing – original draft. MT: Conceptualization, Supervision, Validation, Writing – original draft. EN: Conceptualization, Supervision, Validation, Writing – original draft. CM: Conceptualization, Supervision, Validation, Writing – original draft. MB: Formal analysis, Investigation, Methodology, Writing – original draft. RO: Conceptualization, Supervision, Validation, Writing – original draft. JL-D: Conceptualization, Supervision, Validation, Writing – original draft. PF: Data curation, Formal analysis, Software, Supervision, Writing – original draft. N-MM: Conceptualization, Data curation, Formal analysis, Methodology, Project administration, Resources, Software, Supervision, Validation, Writing – original draft, Writing – review & editing.

## Supplementary material

Supplementary Figure 1 Plan of the maternity and neonatology wards.
