# Supplementary figures and images for: Carbapenem-producing Enterobacteriaceae in mothers and newborns in southeast Gabon, 2022
Source: Front Cell Infect Microbiol. 2024 Feb 8;14:1341161. doi: 10.3389/fcimb.2024.1341161 (PMC10881798; doi:10.3389/fcimb.2024.1341161)

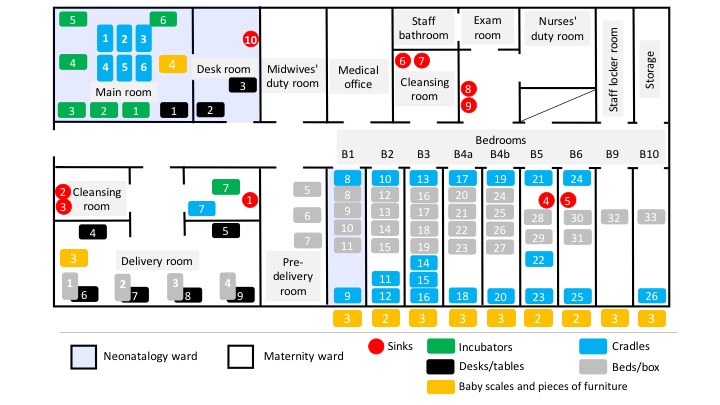

Supplement: Supplementary Figure 1 — Plan of the maternity and neonatology wards. [file Image_1.jpeg]
