# Supplementary material for: Carbapenem-producing Enterobacteriaceae in mothers and newborns in southeast Gabon, 2022
Source: Front Cell Infect Microbiol. 2024 Feb 8;14:1341161. doi: 10.3389/fcimb.2024.1341161 (PMC10881798; doi:10.3389/fcimb.2024.1341161)

**Supplementary table 1.**

SNPs differences between the whole genome sequences of the 25 CPE

**
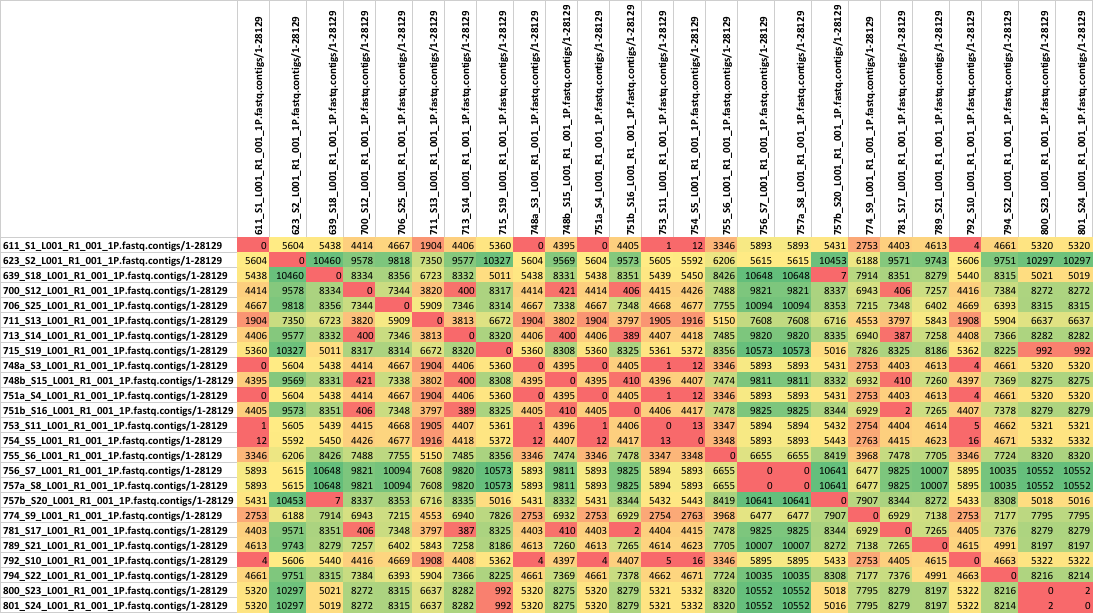
**

**Supplementary table 2.**

Resistome of the 27 carbapenem-resistant strains.

**
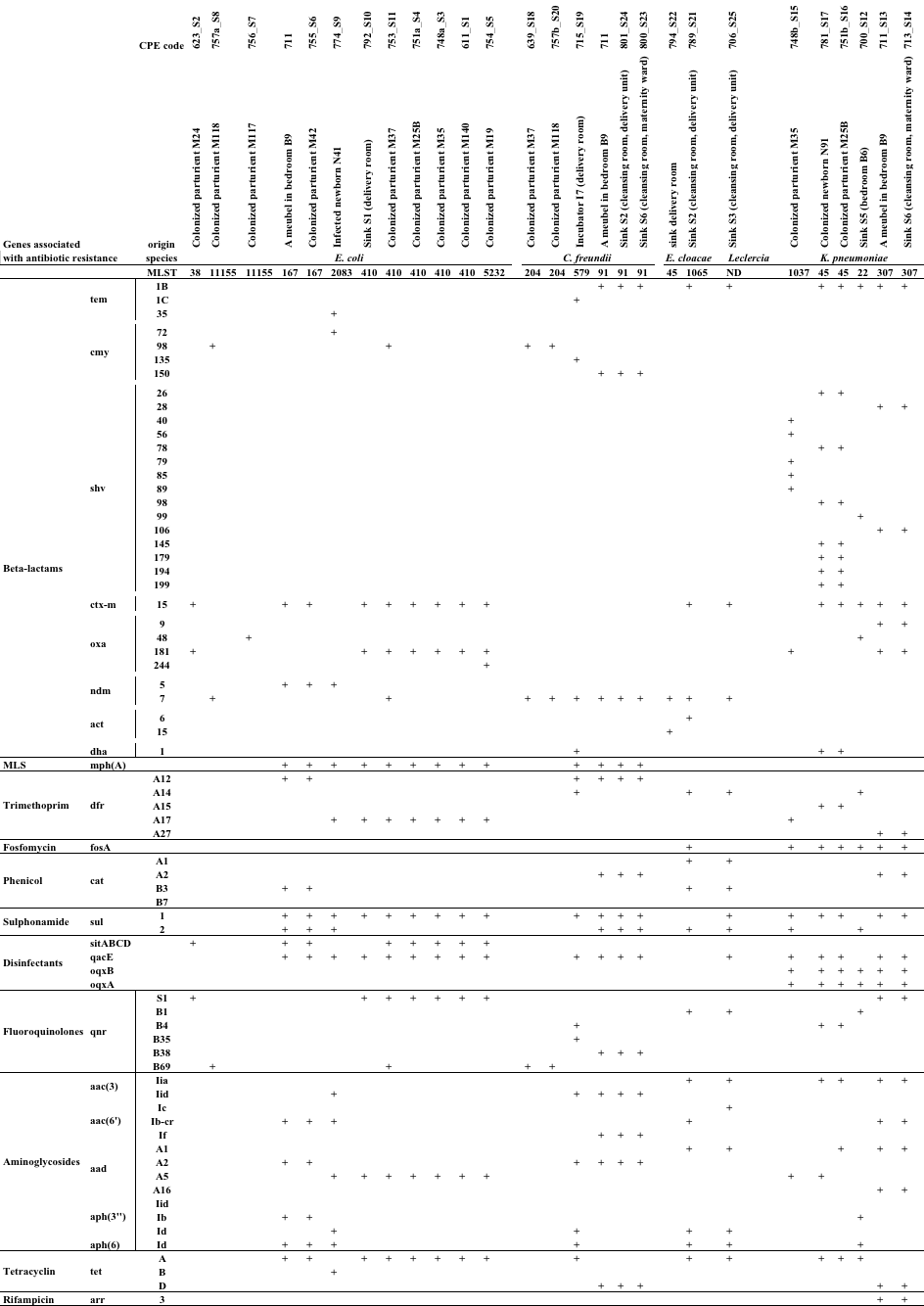
**

**Supplementary table 3.**

Virulome of the 27 carbapenem-resistant strains.


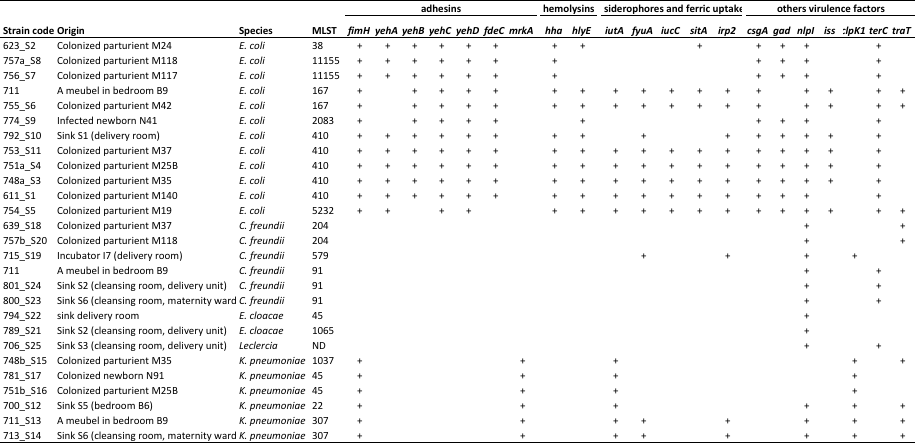

Supplement: Supplementary file 2 [file DataSheet_1.docx]
